# A voice-guided real-time artificial intelligence system for navigation and quality control during standardized mediastinal endosonography scanning

**DOI:** 10.1055/a-2885-8416

**Published:** 2026-10-16

**Authors:** Rui Wang, Shiqin Huang, Xiaoyan Wang

**Affiliations:** 1 Department of Gastroenterology 504354 The Third Xiangya Hospital of Central South UniversityChangshaHunanChina

Endoscopic ultrasound (EUS) plays an important role in mediastinal evaluation because it can access lymph node stations that are not readily reachable by endobronchial ultrasound. 1​ However, its diagnostic quality remains highly operator-dependent, and incomplete recognition of key anatomical landmarks may compromise examination quality control. 2​ ​3​

In this video, we demonstrate a deep learning-based artificial intelligence (AI) system for real-time quality control during mediastinal EUS scanning in a patient undergoing transesophageal mediastinal evaluation ( Video 1 ). The examination is initiated by pressing the “Start mediastinum exam” button on the screen. Once activated, the AI automatically identifies anatomical structures in real time and highlights them with bounding boxes, while simultaneously displaying a checklist of predefined anatomical landmarks and procedural progress on the right side of the screen. In addition, contextual voice prompts with synchronized subtitles guide the endoscopist to the next scanning position, helping reduce blind-spot omission. At the end of the examination, the total quality score and scan duration are displayed in the upper right corner of the screen.

**Video 1** Real-time demonstration of a voice-guided artificial intelligence system for navigation and quality control during standardized mediastinal endoscopic ultrasound scanning.


To our knowledge, this is the first AI-based quality control system for mediastinal EUS to integrate voice-guided navigation with multimodal real-time feedback. By enhancing landmark recognition, route standardization, and completeness monitoring, this system may facilitate real-time process quality control during mediastinal EUS and serve as a practical training aid for less-experienced endoscopists.

Endoscopy_UCTN_Code_TTT_1AS_2AC

## Declaration of GenAI Use

ChatGPT (version 5.2) was applied during manuscript drafting to aid in linguistic editing and stylistic enhancement. No AI tools were involved in producing scientific interpretations, processing experimental data, or deriving conclusions. The entire content has been checked and revised by all authors, who bear full responsibility for the accuracy, reliability, and academic integrity of the study.
